# GAS6 Enhances Repair Following Cuprizone-Induced Demyelination

**DOI:** 10.1371/journal.pone.0015748

**Published:** 2010-12-23

**Authors:** Vladislav Tsiperson, Xiaosong Li, Gary J. Schwartz, Cedric S. Raine, Bridget Shafit-Zagardo

**Affiliations:** 1 Department of Pathology, Albert Einstein College of Medicine, Bronx, New York, United States of America; 2 Department of Medicine, Albert Einstein College of Medicine, Bronx, New York, United States of America; 3 Department of Neuroscience, Albert Einstein College of Medicine, Bronx, New York, United States of America; University of Nebraska, United States of America

## Abstract

Growth arrest-specific protein 6 (gas6) activities are mediated through the Tyro3, Axl, and Mer family of receptor tyrosine kinases. Gas6 is expressed and secreted by a wide variety of cell types, including cells of the central nervous system (CNS). In this study, we tested the hypothesis that administration of recombinant human Gas6 (rhGas6) protein into the CNS improves recovery following cuprizone withdrawal. After a 4-week cuprizone diet, cuprizone was removed and PBS or rhGas6 (400 ng/ml, 4 µg/ml and 40 µg/ml) was delivered by osmotic mini-pump into the corpus callosum of C57Bl6 mice for 14 days. Nine of 11 (82%) PBS-treated mice had abundant lipid-associated debris in the corpus callosum by Oil-Red-O staining while only 4 of 19 (21%) mice treated with rhGas6 had low Oil-Red-O positive droplets. In rhGas6-treated mice, SMI32-positive axonal spheroids and APP-positive deposits were reduced in number relative to PBS-treated mice. Compared to PBS, rhGas6 enhanced remyelination as revealed by MBP immunostaining and electron microscopy. The rhGas6-treated mice had more oligodendrocytes expressing Olig1 in the cytoplasm, indicative of oligodendrocyte progenitor cell maturation. Relative to PBS-treated mice, rhGas6-treated mice had fewer activated microglia in the corpus callosum by Iba1 immunostaining. The data show that rhGas6 treatment resulted in more efficient repair following cuprizone-induced injury.

## Introduction

Gas6, the product of growth arrest-specific gene 6 (*Gas6*), is upregulated during growth arrest [1]–[3], and is expressed and secreted by a wide variety of cell types including neurons [3], [4]. Gas6 is a vitamin K-dependent growth factor and is the ligand for the Tyro3, Axl, and Mer (TAM) receptor tyrosine kinase family [5], [6]. In a concentration-dependent manner, Gas6 induces signaling through TAM receptors and exerts its biological effects [7]–[12]. Gas6 signaling induces cell survival [13], [14], proliferation [15], [16], phagocytosis [17], differentiation [18], and prevents cell apoptosis [19], [20]. Gas6 binds phosphatidylserine and promotes phagocytosis of apoptotic cells [21]. Shed soluble forms of Axl and Mer bind Gas6 and can function as decoy receptors regulating Gas6 bioactivity [22], [23]. In multiple sclerosis (MS) lesions, up-regulation of soluble Axl and Mer negatively correlates with Gas6 expression, suggesting that the dysregulation of protective Gas6 receptor signaling may prolong lesion activity [24].

Several studies *in vitro* and *in vivo* indicate that an interaction between Gas6 and its receptors has physiologic relevance [4], [5]. Our previous studies *in vitro* demonstrated that Gas6 protected human and wild type (WT) rodent oligodendrocytes against insulin withdrawal and TNF-alpha induced cytotoxicity [19]. This protection was lost in cultured oligodendrocytes isolated from Axl knock-out mice, but not from WT or Tyro3 knock-outs. A protective effect was mediated by Akt activation and the effect was blocked by treatment with a PI3 kinase inhibitor suggesting that Gas6/Axl/PI3 kinase/AKT signaling may be important for maintaining oligodendrocyte survival under conditions of stress. In another study, we showed that Axl knock-out mice had delayed recovery and prolonged axonal damage following cuprizone toxicity, supporting the observation that the Axl receptor was important for recovery [25]. Also, Gas6 knock-out mice had fewer mature oligodendrocytes and greater microglial activation following cuprizone toxicity and recovery, indicating that TAM receptor activation and regulation affected multiple CNS cell types [26].

Cuprizone administration into mouse chow induces oligodendrocyte cell death, demyelination, axonal injury and microglial activation in the corpus callosum 3–4 weeks following administration. Upregulation of cytokines and chemokines occurs, along with gliosis, peaking at 4 weeks of cuprizone treatment [27]–[32]. In this study, we tested the hypothesis that direct administration of rhGas6 into the corpus callosum by osmotic mini-pump improves recovery following cuprizone toxicity.

## Materials and Methods

### Animals and cuprizone treatment

C57Bl6J mice were housed in a temperature and humidity-controlled environment with a 12-hr light-dark cycle. Mice were maintained on standard mouse chow and given water *ad libitum* prior to cuprizone treatment. Male 8-week-old mice were fed 0.2% (w/w) cuprizone (bis-cyclohexanone oxalydihydrazone, Sigma; St. Louis, MO) in powdered chow *ad libitum* for 4 weeks. Food containing cuprizone was changed every 2 days for 4 weeks. Immediately after cuprizone withdrawal from the diet, rhGas6 or PBS mini-pump treatment was initiated for an additional 14 days.

### RhGas6 or PBS administration

The Alzet Brain Infusion Kit 3 (Alzet Corp., CA) and the Alzet osmotic mini-pump (Alzet 1002, Alzet Corp., CA), were used to deliver rhGas6 or PBS (vehicle) directly into the corpus callosum. Sterile rhGas6 diluted in PBS was kindly provided by Amgen (Thousand Oaks, CA). The assembly and pre-incubation of the mini-pump were performed according to the manufacturer's instructions. Briefly, one day before surgery, mini-pumps were connected to the brain infusion cannula by catheter tubing and filled with either rhGas6 or PBS. The filled infusion assembly with attached osmotic mini-pump was incubated overnight in sterile saline at 37°C.

RhGas6 was diluted in PBS and three doses, 400 ng/ml (5.2 nM); 4 µg/ml and 40 µg/ml, were administered by mini-pump. PBS was infused as a control. For treatment, mice were anesthetized with a mixture of Ketamine (30mg/kg)/Xylazine (6 mg/kg)/Acepromazine (1 mg/kg), and placed in a stereotaxic frame with non-traumatic ear-bars to hold the skull in place. A 23G needle was used to create a hole above the entry site for the sterile stainless steel mini-pump cannula and the stereotactic arm holding the cannula was lowered into place unilaterally targeting the corpus callosum. The coordinates of the hole for the cannula were 1.0 mm anterior to the bregma, 0.8 mm lateral, and 2.3 mm ventral to the skull surface. The cannula was implanted and glued to the skull surface using veterinary skin adhesive (Loctite 454, Global Test Supply, NC). Thereafter, the cannula was covered by orthodontic powder diluted in acrylic resin liquid (Lang Dental Inc., IL). The mini-pump was inserted beneath the skin at the right scapula and the skin was closed using surgical adhesive. The volume and the rate of delivery of PBS or rhGas6 by mini-pump were 250 nanoliters per hour continuously for 14 days. Mice were housed individually to avoid possible damage to the mini-pump.

All protocols were approved by the Animal Care and Use Committee at the Albert Einstein College of Medicine. The Animal Use Protocol Number is 20080308.

### Tissue preparation, immunohistochemistry and Oil-Red-O staining

After 2 weeks of rhGas6 or PBS treatment, mice were euthanized by cardiac perfusion with 4% paraformaldehyde (PFA). The brains were fixed in 4% PFA for 24 h, cryoprotected in 30% sucrose, and 20 µm frozen sections were prepared and used for analysis from the area corresponding to sections 230–250 of Sidman's Atlas of the mouse brain and spinal cord [33]. To confirm that a significant reduction in myelin occurred in the corpus callosum of mice on the cuprizone diet at 4 weeks, and to examine the extent of remyelination at 14 days following cuprizone withdrawal and rhGas6 or PBS treatment, multiple non-serial coronal sections of mouse brain were stained with myelin basic protein (MBP) monoclonal antibody, SMI99. For immunohistochemistry, all sections were incubated for 30 min with 1× Tris-buffered saline pH 7.4 (1× TBS), containing 0.25% Triton X-100 and 3% H_2_O_2_, followed by a 1 h incubation in 5% goat serum and 5% nonfat dry milk in 1× TBS. Antibodies were diluted in 5% nonfat dry milk in 1× TBS and sections were incubated overnight at 4°C. Following incubation, the sections were washed 3 times in 1× TBS containing 0.1% Triton X-100 and incubated with biotinylated secondary antibody, as detailed in the Vectastain Avidin–Biotin complex kit (Vector Laboratories, Burlingame, CA), and were visualized by 3′3′-diaminobenzidine (DAB, Sigma). The MBP-positive immunostaining was evaluated by Gray value using ImageJ software, with images photographed at a magnification of ×400. This method quantified the extent of MBP expression. Multiple non-serial sections per mouse were used. Data were expressed as mean ± SD.

SMI32, APP, Iba1 and Olig1 immunostaining was performed as described above for MBP. SMI32 and APP are markers of axonal damage [34]-[36]. SMI32-positive axonal swellings and APP-positive deposits were visualized and counted at a magnification of ×400 in the middle and lateral regions of the corpus callosum at the fornix. Three to six non-serial sections per mouse were used and more than 3 mice per group were examined. Data were expressed as mean ± SD. APP positive deposits were counted and expressed as mean ± SD for multiple non-serial sections per mouse and four mice per group. Iba1 was used as a microglial cell marker [37]. Olig1 was used as a marker of oligodendrocyte progenitor maturation. Cytoplasmic Olig1-positive immunostaining was used as a marker of mature oligodendrocytes [38]. Iba1- and Olig1-positive cells were visualized and counted as above. The number of Iba1- and Olig1-positive cells was expressed as mean ± SD for four mice per group.

The lysochrome dye, Oil-Red-O, was used to demonstrate triglycerides and cholesterol esters which associate in particular with lipid deposits in cells and tissues after injury [39]. Frozen sections were incubated in distilled water for 1 min, followed by a 2 min incubation in 100% propylene glycol (Polyscientific; Bay Shore, New York), and transferred to Oil-Red-O (0.5% solution in propylene glycol, Polyscientific; Bay Shore, New York), for 36 h at room temperature. Sections were incubated for 1 min in 85% propylene glycol, lightly stained with hematoxylin and mounted with glycerine jelly mounting medium (Polyscientific; Bay Shore, New York). Oil-Red-O positive deposits were visualized by light microscopy. Multiple, non-adjacent sections per mouse within regions 230–250 of the corpus callosum were evaluated [33].

Once the positive effect of 400 ng/ml and 4 µg/ml of rhGas6 were revealed following Oil-Red-O staining, SMI32 and MBP immunostaining, we focused the remainder of our analysis on the two lower rhGas6 doses. All comparative analyses of MBP, Olig1 and Iba1 immunostaining and for EM focused on the corpus callosum at the midline [40]. Four mice per group were evaluated for APP, Iba1 and Olig1 immunostaining.

### Electron Microscopy (EM)

Following cardiac perfusion with 4% PFA, brain samples were fixed with 2.0% PFA, 2.5% glutaraldehyde and 0.1 M cacodylate buffer (pH 7.4), and postfixed with 1% osmium tetroxide followed by 1% uranyl acetate, dehydrated through a graded series of ethanol and embedded in LX112 resin (LADD Research Industries, Burlington VT). Ultrathin (80 nm) sections were cut on a Reichert Ultracut UCT, stained with 0.01% uranyl acetate followed by lead citrate and viewed in a JEOL 1200EX or an Hitachi HS600. Brain sections within regions 230–250 were taken and oriented such that cross sections of axons within the corpus callosum were obtained. Non-serial sections were evaluated from each mouse (3 mice per group) and at least four fields per mouse were taken at a magnification of ×5000. Image J software was used to calculate axonal diameter, myelin thickness and g-ratios. Non-serial sections were evaluated and 100 axons per mouse were counted. The g-ratio represents the ratio of the diameter of the axon to the diameter of the axon plus myelin. Approximately 14 fields (per animal) with dystrophic axons and axonal spheroids were taken randomly at ×5000 and their sizes were measured in both PBS- and rhGas6-treated mice. All data were reported as the mean ± SD.

#### Antibodies

Both SMI99 (MBP, 1∶1500) and SMI32 (1∶1000) were purchased from Covance Inc, Emeryville, CA. Mouse anti-Alzheimer precursor protein A4 antibody (APP, 22C11, 1∶500), was purchased from Millipore Corp., CA. Iba1 antibody for immunohistochemistry (1∶400) was purchased from WAKO, Richmond, VA. Olig1 antibody (DF389, 1∶1000) was kindly provided by Dr. John Alberta from Dana-Farber Cancer Institute, Boston, MA.

### Statistical analysis

The Kruskal-Wallis statistical analysis was applied (Systat 7.0). P<0.05 was considered significant.

## Results

### rhGas6 reduces Oil-Red-O positive deposits post-cuprizone treatment and withdrawal

**Figure 1:**
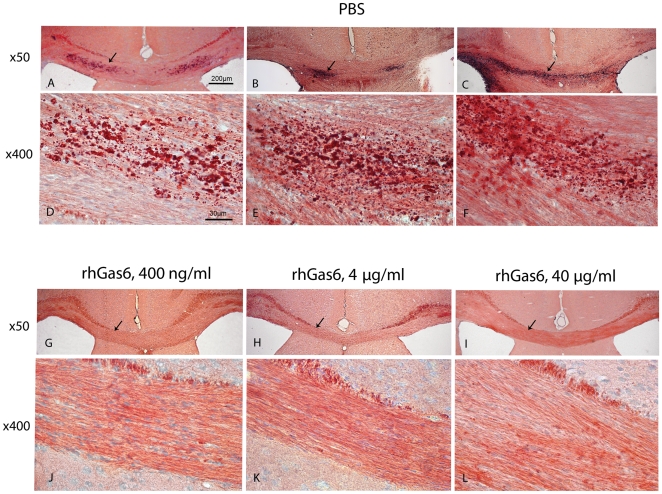
Treatment with rhGas6 reduces Oil-Red-O positive deposits after cuprizone intoxication. A–F, corpus callosum of mouse brain after PBS treatment. Extensive deposition of Oil-Red- O positive droplets can be seen (arrows). Brain sections from mice treated with 400 ng/ml (G and J), 4 µg/ml (H and K), and 40 µg/ml (I and L) of rhGas6 demonstrate a beneficial effect for all tested rhGas6 doses. Arrows indicate the clearance of debris from the corpus callosum. Low and high magnification, ×50 and ×400.

Following treatment with three doses of rhGas6 for 14 days, the corpus callosum of 15 of 19 mice (79%) was completely negative for Oil-Red-O (Fig. 1G-L). Only 4 of 19 mice (21%) treated with 400 ng/ml and 4 µg/ml of rhGas6 had low or moderate staining. By contrast, following PBS treatment, only 2 of 9 mice (18%) were Oil-Red-O negative, whereas 9 of 11 mice (82%) were strongly positive (Fig. 1A–F). Mice fed powdered chow and receiving PBS were Oil-Red-O negative while mice treated with cuprizone for four weeks had extensive positive staining within the corpus callosum (data not shown). These results demonstrated that all rhGas6 doses had a beneficial effect on the clearance of cellular and myelin debris following cuprizone toxicity.

### RhGas6 reduces SMI32+ and APP+ axonal swellings

**Figure 2:**
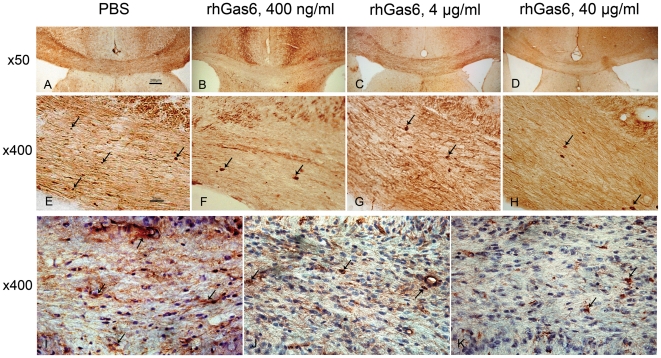
Treatment with rhGas6 improves axonal integrity after cuprizone challenge. SMI32 and APP immunostaining of corpus callosum sections from mice treated for 14 days with PBS (A and E), 400 ng/ml (B and F), 4 µg/ml (C and G), 40 µg/ml of rhGas6 (D and H). Magnification ×50 (A–D), and ×400 (E–K). Increased number of SMI32- positive axonal swellings is observed in the PBS-treated mice (A–E) versus three doses of rhGas6-treated mice (B–H). Arrows show several axonal swellings indicating breakdown in axonal structure. I–K demonstrate APP immunostaining in mice treated with PBS (I), 400 ng/ml (J) and 4 µg/ml of rhGas6 (K). Arrows show APP positive deposits in the corpus callosum, ×400.

Brain sections from both PBS- and rhGas6-treated mice were evaluated for evidence of axonal damage using the monoclonal antibody SMI32 that recognizes a non-phosphoepitope on NF proteins H and M, and an APP monoclonal antibody which detects accumulation of APP due to failed axonal transport. The number of positive axonal swellings was counted as a marker of axonal damage. Representative examples are shown of SMI32 positive axonal swellings in PBS-treated mice (Fig. 2A and E), and in rhGas6-treated mice at the various doses (Fig. 2B–D and F–H). The number of axonal swellings in PBS-treated mice was 27.4±8.8 (n = 11). In mice treated with rhGas6 the number of axonal swellings was 14.9±11.3 (400 ng/ml, p<0.02, n = 7), 10.7±12.1 (4 µg/ml, p<0.01, n = 9), and 14.3±3.7 (40 µg/ml, p<0.05, n = 3). Also, APP immunoreactivity was significantly reduced in rhGas6-treated mice relative to PBS-treated mice (Fig. 2I–K). In PBS-treated mice (n = 4), there were 18.5±6.9 APP positive deposits while in rhGas6-treated mice it was 7.0±2.9 (400 ng/ml, p<0.05, n = 4) and 7.5±3.7 (4 µg/ml, p<0.05, n = 4). Thus, these results showed that all tested doses of rhGas6 significantly reduced the number of axonal swellings relative to the PBS-treated group.

### RhGas6 enhances remyelination after cuprizone toxicity

**Figure 3:**
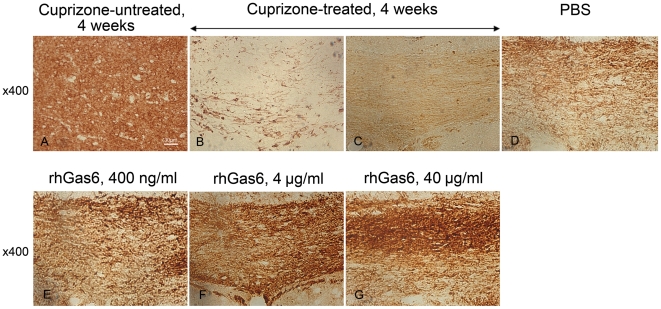
MBP immunostaining shows that rhGas6 treatment enhances remyelination following cuprizone toxicity. Relative to untreated mice (A) there is demyelination of the corpus callosum of mice fed cuprizone for 4 weeks (B and C). A comparison of remyelination assessed by MBP staining of the PBS treated mice (D) versus rhGas6 treatment for 14 days following cuprizone withdrawal (E – 400 ng/ml; F - 4 µg/ml; G – 40 µg/ml of rhGas6) shows that the extent of remyelination is greater in rhGas6-treated mice, ×400.

MBP immunoreactivity was used to estimate the extent of demyelination in the corpus callosum following cuprizone intoxication and to evaluate remyelination after cuprizone withdrawal in the PBS- and rhGas6-treated groups. At 4 weeks post treatment with cuprizone, there was visible demyelination relative to the untreated mice maintained on powdered chow (Fig. 3A–C). The gray value analysis of the intensity of MBP expression confirmed demyelination 4 weeks after cuprizone treatment: 164.4±1.3 (untreated mice, n = 2) vs. 127.4±10.7 (cuprizone-treated mice, n = 3). At 14 days after cuprizone removal and treatment with either PBS or rhGas6, remyelination was observed (Fig. 3D–G). The gray value of PBS-treated mice was 135.5±6.0 versus 140.3±6.6 (400 ng/ml rhGas6, NS, n = 7), 144.9±7.6 (4 µg/ml rhGas6, n = 9, p<0.02) and 142.2±1.9 (40 µg/ml rhGas6, p<0.05, n = 3). Thus, based on gray values, we established that the extent of remyelination was higher in all rhGas6-treated mice versus PBS-treated mice; however, only the 4 µg/ml and 40 µg/ml groups were statistically significant.

### RhGas6 accelerates maturation of oligodendrocyte progenitor cells

**Figure 4:**
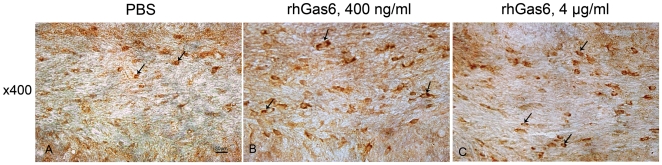
Number of Olig1-positive mature oligodendrocytes is increased in rhGas6-treated mice. Olig1-positive nuclear and cytoplasmic localization in cells (A–C). Increased numbers of cells with Olig1-positive cytoplasmic localization (arrows) in mice treated with 400 ng/ml (B), and 4 µg/ml rhGas6 (C) versus PBS-treated mice (A), are shown, ×400.

Oligodendrocyte progenitor development can be used as a marker of repair after damage caused by cuprizone toxicity. Olig1 is critical for the maturation of oligodendrocyte progenitor cells (OPC), and Olig1 immunostaining becomes progressively more cytoplasmic during OPC maturation. Thus, we used Olig1 cytoplasmic immunostaining as a maturation marker during regeneration. In rhGas6-treated mice, the values were 25.0±18.2 (400 ng/ml, NS, n = 4) and 39.0±7.1 (4 µg/ml, p<0.05, n = 4) while in PBS-treated mice it was 12.0±5.35, n = 4 (Fig. 4). Also, the percentage of cells with Olig1 positive cytoplasmic localization relative to the total number of Olig1 positive cells was increased in rhGas6-treated mice. In PBS-treated mice, the mean percentage was 72.8% ±7.4. The mean percentage was 89.2% ±3.7 in 400 ng/ml rhGas6-treated mice and 96.6% ±1.8 in 4 µg/ml rhGas6-treated mice. Therefore, these findings showed that the number of cells with Olig1-positive cytoplasmic localization was increased in rhGas6- treated mice.

### Number of myelinated axons is increased in mice treated with rhGas6

**Figure 5:**
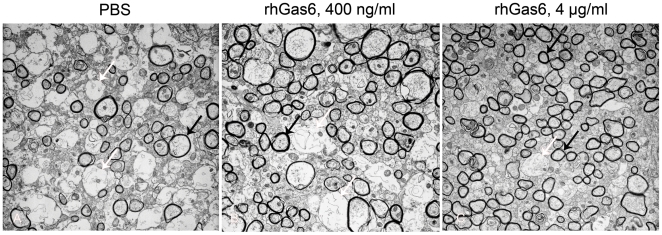
EM shows more myelinated axons in rhGas6-treated mice. Relative to PBS (A), the number of myelinated axons was significantly increased in mice treated with 400 ng/ml (B) and 4 µg/ml (C) rhGas6. Black arrows denote myelinated axons; white arrows show demyelinated, naked axons, ×5000.

EM was performed to determine whether the increased MBP immunoreactivity and enhanced maturation of oligodendrocytes in the rhGas6-treated groups of mice reflected more myelinated axons in the corpus callosum. More structured tissue architecture, organized axons as well as more myelinated axons in rhGas6-treated mice relative to PBS-treated mice were observed (Fig. 5). While administration of 400 ng/ml rhGas6 increased the number of myelinated axons (Fig.5B), it was not statistically significant relative to PBS treatment (Table 1). The number of myelinated axons was significantly increased (p<0.02) in mice treated with 4 µg/ml rhGas6 (Fig. 5C). Axonal diameter was smaller in mice treated with rhGas6- versus PBS-treated mice, whereas only 4 µg/ml rhGas6 was statistically significant. The g-ratio was significantly decreased in rhGas6- versus PBS-treated mice, indicating increased myelin thickness in rhGas6-treated mice. These data showed that relative to PBS treatment, administration of rhGas6 enhanced remyelination in the corpus callosum 14 days following treatment.

**Table 1:** Increased number of myelinated axons is observed in mice treated with rhGas6.

Parameters	PBS	rhGas6400 ng/ml	rhGas64 µg/ml
Number of myelinated axons, mean ± SD^a^	48.0±20.1	80.6±6.6	100.5±18.6**
* g-ratio, mean ± SDa, b	0.877±0.004	0.853±0.007**	0.836±0.01**
Axonal diameter,mean ± SD^b^	0.868±0.063	0.752±0.057	0.70±0.067**

a - data was collected from four photomicrographs, ×5000; 3 mice/group.

b - 100 randomly selected axons were measured per mouse.

*- g-ratio of cuprizone untreated mice was 0.802±0.01 (mean ±s SD).

**- P<0.05.

**Figure 6:**
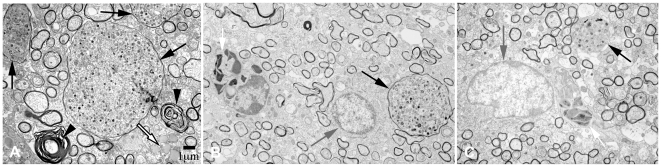
EM demonstrates dystrophic axons and axonal spheroids in PBS- and rhGas6-treated mice. A. Axonal spheroids (>10 µm) are shown in PBS-treated mice (black arrow). Myelin debris (arrowhead) and lipid droplets (white arrow), are also noted. B – 400 ng/ml, C – 4 µg/ml of rhGas6 demonstrate dystrophic axons ranging in size from 3.0–9.5 µm in diameter (black arrow). Note microglial cell process (white arrow, B), and astrocytes in rhGas6-treated mice (gray arrow B and C), ×5000.

To determine whether axonal swellings detected by SMI32 immunostaining constituted dystrophic axons or spheroids (10–50 µm), we measured the size of axonal swellings in mice treated with PBS and rhGas6. Dystrophic axons ranged from 3.0 µm to 9.5 µm in diameter while axonal spheroids were greater than 10.0 µm in diameter (Fig. 6). The percentage of axonal spheroids and dystrophic axons was calculated from the total number of injured axons from approximately 14 randomly selected fields at a magnification ×5000. The percentage of smaller dystrophic axons was higher in rhGas6- versus PBS-treated mice: 77% (400 ng/ml rhGas6); 86% (4 µg/ml rhGas6) compared to 42% (PBS). By contrast, the percentage of axonal spheroids was greater in PBS-treated relative to rhGas6-treated mice: 23% (400 ng/ml rhGas6); 14% (4 µg/ml rhGas6) compared to 58% (PBS). In addition, EM revealed more myelin and lipid debris in PBS-treated relative to rhGas6-treated mice. Representative micrograph (Fig. 6A) shows myelin debris and lipid deposits in PBS-treated mouse. Therefore, EM supported our observation that rhGas6 administration had a positive effect on the clearance of debris.

### Number of activated microglial cells not significantly altered after PBS or rhGas6 treatment

**Figure 7:**
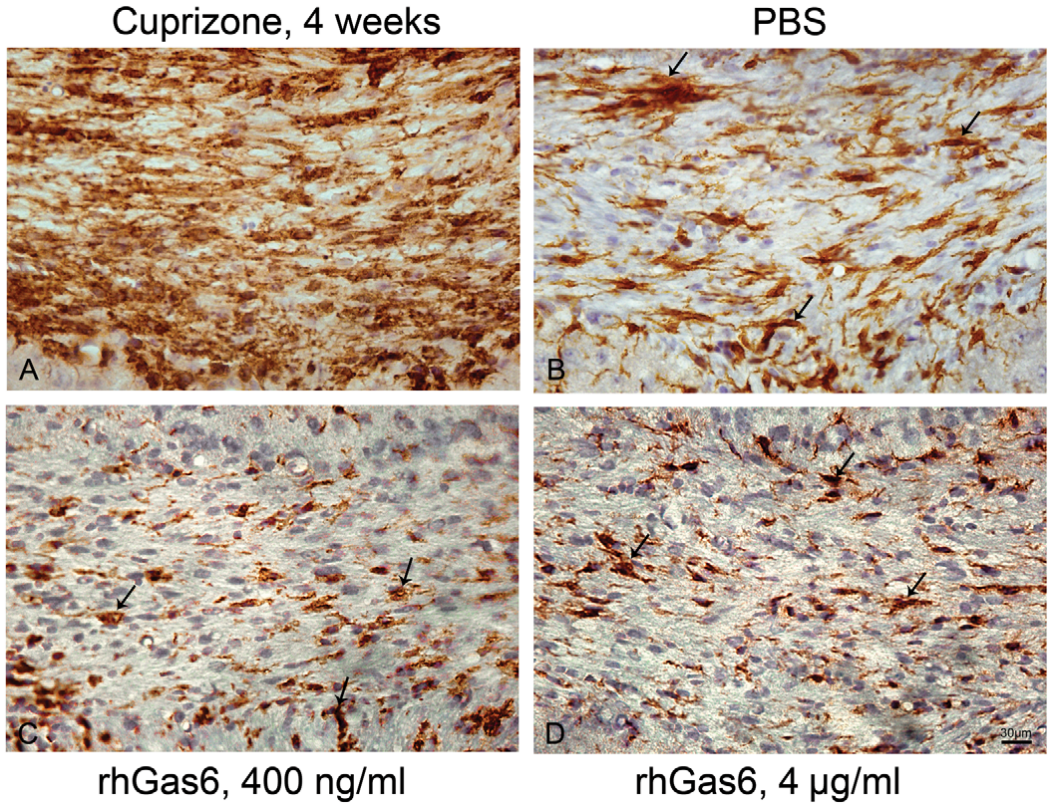
Similar numbers of Iba1-positive microglia are present in PBS- and rhGas6-treated mice. A. Following cuprizone ingestion for 4 weeks an extensive Iba1-positive microglial activation within the corpus callosum is observed. There was significant reduction of Iba1-postive microglia in both PBS- (B), and rhGas6-treated mice (C and D) at 14 days post treatment relative to 4 week cuprizone treated mice (p<0.02). Arrows show Iba1-positive microglia.

We used Iba1 as a marker of microglial cell activation to determine whether following direct delivery of rhGas6 versus PBS, there was a reduction in Iba1-positive immunoreactivity. Representative Iba1-positive microglia immediately following cuprizone treatment for 4 weeks is shown (Fig. 7). Abundant Iba1-positive microglia (92.0±4.2, n = 4) was seen immediately following 4 weeks of cuprizone treatment. Significant reduction in Iba1-positive microglial cells at 14 days following cuprizone withdrawal and treatment with either PBS or rhGas6 was observed. The number of Iba1-positive cells in the rhGas6-treated mice was 38.0±14.6, 400 ng/ml, (n = 4), and 34.0±3.7, 4 µg/ml, (n = 4) relative to PBS-treated mice 48.0±17.7, (n = 4), the differences were not statistically significant.

Overall, these results suggest that compared to PBS, rhGas6 treatment resulted in more efficient repair of the corpus callosum following cuprizone toxicity. This conclusion is based upon enhanced clearance of cellular and myelin debris and remyelination, increased axonal survival and integrity as well as OPC maturation.

## Discussion

Gas6 is a survival factor for neurons and glial cells involved in myelination as well as a growth and anti-apoptotic factor for Schwann cells [41]. Gas6 and the TAM receptors are widely expressed in the rodent nervous system [4]. In this study, we show that compared to mice receiving PBS, rhGas6 administration directly to the corpus callosum by osmotic mini-pump for 14 days enhanced the clearance of lipid-laden debris, remyelination and reduced axonal damage that resulted from 4 weeks of ingestion of cuprizone in powdered chow. Indeed, 14 days after treatment, nine of eleven PBS-treated mice had extensive lipid-associated droplets in the corpus callosum and only four of nineteen mice treated with multiple rhGas6 doses had low or moderate Oil-Red-O positive staining. These data demonstrated that *in vivo*, rhGas6 enhanced the clearance of myelin debris which was efficiently eliminated after cuprizone damage. In another study, in which lysolecithin was injected directly into the cervical spinal cord, large deposits of Oil-Red-O were observed, correlating with areas of demyelination 7–14 days after injection. Despite the observation that the density of the lipid-laden droplets inside the lesion area decreased over the time course examined, it did not disappear entirely [42]. Perhaps, rhGas6 can be considered as a potential enhancer of debris clearance and remyelination following spinal cord injury.

Although the number of activated microglia in PBS- relative to rhGas6-treated mice was not significantly different, rhGas6 likely was more effective for clearance of debris. A positive effect of Gas6 on dying cell clearance *in vitro* has been reported for multiple cell types [43]–[46], and the ability of rhGas6 to affect phagocytosis in murine microglia by acting on Axl/Mer has been shown previously *in vitro* [47].

The clearance of cellular and myelin debris might be an initial and important early step for recovery in the corpus callosum. Efficient clearance of damaged cells and myelin debris likely impacts upon remyelination and cell survival. In this study, a significantly greater number of SMI32-positive axonal spheroids were observed in PBS- versus rhGas6-treated mice. After treatment with different doses of rhGas6, all doses noticeably reduced the number of SMI32 positive axonal swellings relative to PBS treatment. SMI32-positive immunoreactivity in axons as well as the presence of axonal swellings or spheroids is considered pathologic and was previously shown to occur during cuprizone treatment and during MOG-induced experimental autoimmune encephalomyelitis [34], [35], [48], [49].

Increased SMI32-positive immunoreactivity correlates with axonal injury due to dephosphorylation of neurofilament H [25], [50]. Further, SMI32 immunoreactivity is found in chronic lesions from patients with MS [51]. Similarly, increased APP immunoreactivity within axons represents a defect in axonal transport and serves as a marker of axonal injury resulting from cytoskeletal breakdown and calcium influx that interrupts axoplasmic flow and subsequent accumulation of organelles [36]. By EM, we showed that the percentage of small dystrophic axons was greater in mice treated with rhGas6 whereas the percentage of large axonal spheroids was higher in PBS-treated mice relative to rhGas6. Thus, data from EM support our conclusion that mice treated with rhGas6 have a reduction in axonal spheroids. The results obtained demonstrate a beneficial effect of rhGas6 on axonal survival and the maintenance of axon integrity following cuprizone toxicity. In this context, we previously showed *in vitro* that 400 ng/ml (5.2 nM) rhGas6 had a maximal positive effect on human oligodendrocyte survival using oligodendrocytes isolated from human fetal spinal cord. This suggests that rhGas6 serves as a survival factor for oligodendrocytes during development [52].

In the present study, we evaluated how rhGas6 affected remyelination following cuprizone withdrawal by MBP immunostaining. We used gray value analysis to distinguish the intensity of MBP expression between PBS- and rhGas6-treated mice. Extensive demyelination in the corpus callosum (Fig. 3B and C), was observed after 4 weeks of cuprizone treatment. The extensive myelin loss had the lowest gray value which correlated with weak MBP immunoreactivity. Our data are consistent with other studies demonstrating demyelination in the corpus callosum 3–4 weeks after cuprizone treatment [53], [54]. Quantification of MBP staining revealed that gray values gradually increased after cuprizone removal in both PBS- and rhGas6-treated mice. The increase in gray values likely reflected progressive remyelination consistent with the MBP expression. Comparative analysis of gray values in rhGas6- versus PBS-treated mice demonstrated that 4 µg/ml and 40 µg/ml of rhGas6 were more effective for remyelination compared to the 400 ng/ml rhGas6. Although the dose of 400 ng/ml of rhGas6 did not produce statistically significant results, these mice also had more MBP immunoreactivity than PBS-treated mice, indicating that enhanced clearance of debris and reduced number of axonal spheroids was associated with successful remyelination. EM showed increased numbers of myelinated axons and a decreased g-ratio in rhGas6-treated mice indicative of increased myelin thickness. Thus, the data from EM are consistent with results obtained by MBP immunostaining. It was shown that following acute demyelination after cuprizone intoxication, many small caliber axons become preferentially remyelinated [30]. We also found that the number of small diameter myelinated axons was significantly increased in rhGas6-treated mice. Thus, remyelination of small axons was an important index of corpus callosum recovery.

As OPCs mature, they synthesize myelin proteins that contribute to remyelination. We used Olig1 immunostaining as a marker of OPC maturation to determine if direct administration of rhGas6 affected OPC development. Olig1, a closely related homolog to Olig2, is co-expressed with Olig2 in many cells of the oligodendrocyte lineage. As OPCs mature, there is a shift in Olig1 immunostaining from a nuclear to a cytoplasmic localization [38]. Analysis of the corpus callosum showed that the number of cells with Olig1-positive cytoplasmic localization was increased in rhGas6-treated mice but only the dose of 4 µg/ml was statistically significant. Further, the percentage of cells with Olig1-positive cells with cytoplasmic localization relative to total number of Olig1-positive cells was increased in mice treated with 4 µg/ml of rhGas6.

Taken together, these data provide compelling support for rhGas6 having a beneficial effect on corpus callosum recovery following cuprizone toxicity. Although 400 ng/ml rhGas6 was a therapeutic dose for the clearance of cellular debris, maintenance of cell survival and axonal integrity, 4 µg/ml of rhGas6 was more effective for remyelinationy. To our knowledge this is the first report of a beneficial effect of rhGas6 administration *in vivo*. These results open new avenues for rhGas6 as a treatment modality, in addition to the study of mechanisms by which rhGas6 exerts this effect.

## References

[1] Schneider C, King RM, Philipson L (1988). Genes specifically expressed at growth arrest of mammalian cells.. Cell.

[2] Manfioletti G, Brancolini C, Avanzi G, Schneider C (1993). The protein encoded by a growth arrest-specific gene (gas6) is a new member of the vitamin K-dependent proteins related to protein S, a negative coregulator in the blood coagulation cascade.. Mol Cell Biol.

[3] Saller F, Burnier L, Schapira M, Angelillo-Scherrer A (2006). Role of the growth arrest-specific gene 6 (gas6) product in thrombus stabilization.. Blood Cells Mol Dis.

[4] Prieto A, Weber JL, Lai C (2000). Expression of the receptor protein tyrosine kinase Tyro3, Axl and Mer in the developing rat central nervous system.. J Comp Neurol.

[5] Li R, Chen J, Hammonds G, Phillips H, Armanini M (1996). Identification of Gas6 as a growth factor for human Schwann cells.. J Neurosci.

[6] Hafizi S, Dahlback B (2006). Gas6 and protein S. Vitamin K-dependent ligands for the Axl receptor tyrosine kinase subfamily.. FEBS J.

[7] Stitt TN, Conn G, Gore M, Lai C, Bruno J (1995). The anticoagulation factor protein S and its relative, Gas6, are ligands for the Tyro 3/Axl family of receptor tyrosine kinases.. Cell.

[8] Ohashi K, Nagata K, Toshima J, Nakano T, Arita H (1995). Stimulation of sky receptor tyrosine kinase by the product of growth arrest-specific gene 6.. J Biol Chem.

[9] Nagata K, Ohashi K, Nakano T, Arita H, Zong C (1996). Identification of the product of growth arrest-specific gene 6 as a common ligand for Axl, Sky, and Mer receptor tyrosine kinases.. J Biol Chem.

[10] Mark M, Chen J, Hammonds RG, Sadick M, Godowski PJ (1996). Characterization of Gas6, a member of the superfamily of G domain-containing proteins, as a ligand for Rse and Axl.. J Biol Chem.

[11] Gould WR, Baxi SM, Schroeder R, Peng YW, Leadley RJ (2005). Gas6 receptors Axl, Sky and Mer enhance platelet activation and regulate thrombotic responses.. J Thromb Haemost.

[12] Gallicchio M, Mitola S, Valdembri D, Fantozzi R, Varnum B (2005). Inhibition of vascular endothelial growth factor receptor 2-mediated endothelial cell activation by Axl tyrosine kinase receptor.. Blood.

[13] Allen MP, Zeng C, Schneider K, Xiong X, Meintzer MK (1999). Growth arrest-specific gene 6 (Gas6)/adhesion related kinase (Ark) signaling promotes gonadotropin-releasing hormone neuronal survival via extracellular signal-regulated kinase (ERK) and Akt.. Mol Endocrinol.

[14] Yagami T, Ueda K, Asakura K, Okamura N, Sakaeda T (2003). Effect of Gas6 on secretory phospholipase A(2)-IIA-induced apoptosis in cortical neurons.. Brain Res.

[15] Yanagita M, Ishimoto Y, Arai H, Nagai K, Ito T (2002). Essential role of Gas6 for glomerular injury in nephrotoxic nephritis.. J Clin Invest.

[16] Nakano T, Kawamoto K, Kishino J, Nomura K, Higashino K (1997). Requirement of c-carboxyglutamic acid residues for the biological activity of Gas6: contribution of endogenous Gas6 to the proliferation of vascularsmooth muscle cells.. Biochem J.

[17] Hall MO, Obin MS, Prieto AL, Burgess BL, Abrams TA (2002). Gas6 binding to photoreceptor outer segments requires gamma-carboxyglutamic acid (Gla) and Ca(2+) and is required for OS phagocytosis by RPE cells in vitro.. Exp Eye Res.

[18] Caraux A, Lu Q, Fernandez N, Riou S, Di Santo JP (2006). Natural killer cell differentiation driven by Tyro3 receptor tyrosine kinases.. Nat Immunol.

[19] Shankar SL, O'Guin K, Kim M, Varnum B, Lemke G (2006). Gas6/Axl signaling activates the phosphatidylinositol 3-kinase/Akt1 survival pathway to protect oligodendrocytes from tumor necrosis factor alpha-induced apoptosis.. J Neurosci.

[20] Hasanbasic I, Rajotte I, Blostein M (2005). The role of gamma-carboxylation in the anti-apoptotic function of gas6.. J Thromb Haemost.

[21] Wu Y, Singh S, Georgescu MM, Birge RB (2005). A role for Mer tyrosine kinase in αvβ5 integrin-mediated phagocytosis of apoptotic cells.. J Cell Sci.

[22] Sather S, Kenyon KD, Lefkowitz JB, Liang X, Varnum BC (2007). A soluble form of the Mer receptor tyrosine kinase inhibits macrophage clearance of apoptotic cells and platelet aggregation.. Blood.

[23] Budagian V, Bulanova E, Orinska Z, Duitman E, Brandt K (2005). Soluble Axl is generated by ADAM10-Dependent cleavage and associates with Gas6 in mouse serum.. Mol Cell Biol.

[24] Weinger JG, Omari KM, Marsden M, Raine CS, Shafit-Zagardo B (2009). Up-regulation of soluble Axl and Mer receptor tyrosine kinases negatively correlates with Gas6 in established multiple sclerosis lesions.. Am J Pathol.

[25] Hoehn HJ, Kress Y, Sohn A, Brosnan CF, Bourdon S (2008). Axl−/− mice have delayed recovery and prolonged axonal damage following cuprizone toxicity.. Brain Res.

[26] Binder MD, Cate HS, Prieto AL, Kemper D, Butzkueven H (2008). Gas6 deficiency increases oligodendrocyte loss and microglial activation in response to cuprizone-induced demyelination.. J Neurosci.

[27] Blakemore WF (1973). Demyelination of the superior cerebellar peduncle in the mouse induced by cuprizone.. J Neurol Sci.

[28] Blakemore WF (1973). Remyelination of the superior cerebellar peduncle in the mouse following demyelination induced by feeding cuprizone.. J Neurol Sci.

[29] Hiremath MM, Saito Y, Knapp GW, Ting JPY, Suzuki K (1998). Microglial/macrophage accumulation during cuprizone-induced demyelination in C57BL/6 mice.. J Neuroimmunol.

[30] Mason JL, Langaman C, Morell P, Suzuki K, Matsushima GK (2001). Episodic demyelination and subsequent remyelination within the murine central nervous system: changes in axonal caliber.. Neuropathol Appl Neurobiol.

[31] Matsushima GK, Morell P (2001). The neurotoxicant, cuprizone, as a model to study demyelination and remyelination in the central nervous system.. Brain Pathol.

[32] Jurevics H, Largent C, Hostettler J, Sammond DW, Matsushima GK (2006). Alterations in metabolism and gene expression in brain regions during cuprizone-induced demyelination and remyelination.. J Neurochem.

[33] Sidman RL, Angevine JB, Pierce EB Atlas of the Mouse Brain and Spinal Cord..

[34] Stidworthy MF, Genoud S, Suter U, Mantei N, Franklin (2003). Quantifying the early stages of remyelination following cuprizone-induced demyelination.. Brain Pathol.

[35] Irvine KA, Blakemore WF (2006). Age increases axon loss associated with primary demyelination in cuprizone-induced demyelination in C57BL/6 mice.. J Neuroimmunol.

[36] Ferguson B, Matyszak MK, Esiri MM, Perry VH (1997). Axonal damage in acute multiple sclerosis lesions.. Brain.

[37] Okebe CO, Kaba H (2000). Heterogenous immunohistochemical expression of microglia-specific ionized calcium binding adaptor protein (Iba1) in the mouse olfactory bulb.. Brain Res.

[38] Arnett HA, Fancy SPJ, Alberta JA, Zhao C, Plant SR (2004). bHLH transcription factor Olig1 is required to repair demyelinated lesions in the CNS.. Science.

[39] Barbarese E, Ho SY, D'Arrigo JS: Simon R (1995). Internalization of microbubbles by tumor cells *in vivo* and *in vitro.*. J Neurooncol.

[40] Mason JL, Ye P, Suzuki K, D'Ercole AJ, Matsushima GK (2000). Insulin-like growth factor-1 inhibits mature oligodendrocyte apoptosis during primary demyelination.. J Neurosci.

[41] Sainaghi PP, Collimedaglia L, Alciato F, Leone MA, Puta E (2008). Elevation of Gas6 protein concentration in cerebrospinal fluid of patients with chronic inflammatory demyelinating polyneuropathy (CIDP).. J Neurol Sci.

[42] McCreary CR, Bjarnason TA, Skihar V, Mitchell JR, Yong (2009). Multiexponential T2 and magnetization transfer MRI of demyelination and remyelination in murine spinal cord.. Neuroimage.

[43] Ishimoto Y, Ohashi K, Mizuno K, Nakano T (2000). Promotion of the uptake of PS liposomes and apoptotic cells by a product of growth arrest-specific gene, gas6.. J Biochem.

[44] Yagami T, Ueda K, Asakura K, Sakaeda T, Nakazato H (2002). Gas6 rescues cortical neurons from β amyloid protein-induced apoptosis.. Neuropharmacol.

[45] Hall MO, Agnew BJ, Abrams TA, Burgess BL (2003). The phagocytosis of outer segments is mediated by the PI3-kinase linked tyrosine kinase receptor, mer, and is stimulated by Gas6.. Adv Exp Med Biol.

[46] Xiong W, Chen Y, Wang H, Wang H, Wu H (2008). Gas6 and the Tyro 3 receptor tyrosine kinase subfamily regulate the phagocytic function of Sertoli cells.. Reproduction.

[47] Grommes C, Lee CYD, Wilkinson BL, Jiang Q, Koenigsknecht-Talboo JC (2008). Regulation of microglial phagocytosis and inflammatory gene expression by Gas6 acting on the Axl/Mer family of tyrosine kinases.. J Neuroim Pharmacol.

[48] Song SK, Yoshino J, Le TQ, Lin SJ, Sun SW (2005). Demyelination increases radial diffusivity in corpus callosum of mouse brain.. Neuroimage.

[49] Herrero-Herranz E, Pardo LA, Gold R, Linker RA (2008). Pattern of axonal injury in murine myelin oligodendrocyte glycoprotein induced experimental autoimmune encephalomyelitis: implications for multiple sclerosis.. Neurobiol Dis.

[50] Jackson SJ, Pryce G, Diemel LT, Cuzner ML, Baker D (2005). Cannabinoid-receptor 1 null mice are susceptible to neurofilament damage and caspase 3 activation.. Neuroscience.

[51] Petzold A, Gveric D, Groves M, Schmierer K, Grant D (2008). Phosphorylation and compactness of neurofilaments in multiple sclerosis: indicators of axonal pathology.. Exp Neurol.

[52] Shankar SL, O'Guin K, Cammer M, McMorris FA, Stitt TN (2003). Basch RS, Varnum B, Shafit-Zagardo B: The growth arrest specific gene product Gas6 promotes the survival of human oligodendrocytes via a phosphatidylinositol 3-kinase-dependent pathway.. J Neurosci.

[53] Gao X, Gillig TA, Ye P, D'Ercole AJ, Matsushima GK (2000). Interferon-γ protects against cuprizone-induced demyelination.. Mol Cell Neurosci.

[54] Lindner M, Heine S, Haastert K, Garde N, Fokuhl J (2008). Sequential myelin protein expression during remyelination reveals fast and efficient repair after central nervous system demyelination.. Neuropathol Appl Neurobiol.

